# Bee Venom Soluble Phospholipase A2 Exerts Neuroprotective Effects in a Lipopolysaccharide-Induced Mouse Model of Alzheimer’s Disease *via* Inhibition of Nuclear Factor-Kappa B

**DOI:** 10.3389/fnagi.2019.00287

**Published:** 2019-11-01

**Authors:** Hyeon Joo Ham, Ji Hye Han, Yong Sun Lee, Ki Cheon Kim, Jaesuk Yun, Shin Kook Kang, YangSu Park, Se Hyun Kim, Jin Tae Hong

**Affiliations:** ^1^College of Pharmacy and Medical Research Center, Chungbuk National University, Chungbuk, South Korea; ^2^INISTst Company Limited, Gyeonggi-do, South Korea

**Keywords:** neuroinflammation, Alzheimer’s disease, NF-κB, regulatory T cells, bee venom phospholipase A2

## Abstract

Neuroinflammation is important in the pathogenesis and development of Alzheimer’s disease (AD). In the AD brain, microglial activation and upregulation of pro-inflammatory mediators both induce amyloid beta (Aβ) accumulation. Regulatory T cells (Tregs) and nuclear factor-kappa B (NF-κB) signaling have been implicated in AD development through their effects on neuroinflammation and microglial activation. The bee venom soluble phospholipase A2 (bv-sPLA2) enzyme is known to exert anti-inflammatory and anti-immune effects. Here, we investigated the inhibitory effects of bv-sPLA2 on memory deficiency in a lipopolysaccharide (LPS)-induced mouse model of AD. We examined whether bv-sPLA2 (0.02, 0.2, and 2 mg/kg by i.p. injection three times for 1 week) could inhibit neuroinflammation and memory impairment in LPS-treated mice (250 μg/kg by i.p. injection daily for 1 week). We also assessed the effects of bv-sPLA2 administration (0.01, 0.1, and 1 μg/ml) on LPS (1 μg/ml)-treated microglial BV-2 cells. In the LPS-injected mouse brain, sPLA2 treatment rescued memory dysfunction and decreased Aβ levels, through the downregulation of amyloidogenic proteins, and decreased the expression of inflammatory proteins and pro-inflammatory cytokines. Moreover, the LPS-mediated increase in inflammatory protein expression was attenuated bv-sPLA2 treatment in BV-2 cells. Treatment with bv-sPLA2 also downregulated signaling by NF-κB, which is considered to be an important factor in the regulation of neuroinflammatory and amyloidogenic responses, both *in vivo* and *in vitro*. Additionally, co-treatment with NF-κB (5 μM) and bv-sPLA2 (0.1 μg/ml) exerted more marked anti-inflammatory effects, compared to bv-sPLA2 treatment alone. These results indicate that bv-sPLA2 inhibits LPS-induced neuroinflammation and amyloidogenesis *via* inhibition of NF-κB.

## Introduction

Amyloid β (Aβ) can accumulate as insoluble, extracellular senile plaque deposits, neuropathological hallmarks of Alzheimer’s disease (AD), which is characterized clinically by cognition and memory impairments (Heppner et al., 2015). The Aβ peptide is generated through the cleavage of the amyloid precursor protein (APP) by gamma- and beta-secretases in the cerebral cortex and hippocampus (Heneka et al., 2015b; Heppner et al., 2015). Accumulating evidence suggests that AD pathogenesis is not restricted to the neuronal compartment, but is also associated with immunological processes in the brain (Heneka et al., 2015a). In the AD brain, Aβ deposition is strongly associated with neuroinflammation and memory dysfunction (Cameron and Landreth, 2010). Microglia are major immune effector cells in the central nervous system (Jung et al., 2009). When activated, microglial cells release high levels of pro-inflammatory mediators, including macrophage inflammatory proteins, prostaglandins, and cytokines like tumor necrosis factor (TNF), interleukin 1β (IL-1β), and IL-6 (Cameron and Landreth, 2010; Rubio-Perez and Morillas-Ruiz, 2012), whichupregulate beta-secretase expression and activity (Sastre et al., 2003).

Systemic treatment with lipopolysaccharides (LPS), the endotoxins produced by gram-negative bacteria, upregulates pro-inflammatory reactions and leads to memory dysfunction (Cui et al., 2008; Zarifkar et al., 2010; Badshah et al., 2016). Astrocytes and microglial cells are activated in the LPS-treated mouse brain (Tanaka et al., 2006; Badshah et al., 2016). Moreover, LPS treatment also upregulates iNOS expression in astrocytes and microglia, and induces the release of neuroinflammatory cytokines that promote amyloidogenesis through increased beta-secretase activity (Heneka and Feinstein, 2001; Jung et al., 2009; Badshah et al., 2016). Neuroinflammation induced by LPS treatment increases both APP expression and beta-secretase activity, thus directly influencing Aβ production (Sheng et al., 2003). Consequently, an increasing number of studies have used LPS-induced models to investigate AD (Cui et al., 2008; Zarifkar et al., 2010; Badshah et al., 2016; Choi et al., 2017a).

Nuclear factor-kappa B (NF-κB), a member of the Rel family of transcription factors, plays critical roles in various inflammatory and autoimmune diseases, including AD. Expression of inflammatory proteins, such as iNOS and cyclooxygenase 2 (COX-2), can be regulated by NF-κB activation (Jung et al., 2009). An increasing number of studies have demonstrated that NF-κB activation, accompanied by secretion of pro-inflammatory cytokines, is associated with neuronal degeneration in the brains of AD patients (Boissière et al., 1997; He et al., 2011). In addition, the NF-κB complex is also involved in amyloidogenensis, as evidenced by the presence of a functional NF-κB binding site in the promoter region of the beta-site APP-cleaving enzyme 1 gene (*BACE1*, Sambamurti et al., 2004). Examination of postmortem brain tissues from AD patients revealed increased NF-κB immunoreactivity in both astrocytes and microglia (Mattson and Camandola, 2001; Paris et al., 2007). These data suggest that downregulation of NF-κB may exert beneficial anti-AD effects by reducing neuroinflammation and amyloidogenensis (Kumar et al., 2004).

The enzyme phospholipase A2 (PLA2) catalyzes the hydrolysis of the sn-2 position of membrane glycerophospholipids, leading to the production of free fatty acids and lysophospholipids (Kudo and Murakami, 2002; Burke and Dennis, 2009). The PLA2 enzyme family has been categorized into three major groups, namely, group IV calcium-dependent cytosolic PLA2 (cPLA2), group II secretory PLA2 (sPLA2), and group VI Ca^2+^-independent PLA2 (iPLA2; Kudo and Murakami, 2002; Sun et al., 2004). Secretory PLA2 is found in the venom of both vertebrate and invertebrate animals, such as snakes, bees and scorpions (Valentin et al., 2000). In mammals, different PLA2 enzymes have been shown to downregulate physiological events, related to cell injury, inflammation, and apoptosis (Sun et al., 2004). Moreover, sPLA2 inhibits phospholipid metabolism, signal transduction, and inflammatory and immune responses (Hanasaki and Arita, 2002; Kudo and Murakami, 2002). Among the different PLA2 groups, sPLA2 has been suggested as an important therapeutic target for a variety of neurodegenerative diseases because it exacerbated autoimmune or inflammatory reactions (Sun et al., 2004; Cunningham et al., 2006; Chen S. et al., 2012). However, bv-sPLA2 has also been reported to exert protective effects on cisplatin-induced kidney injury (Kim et al., 2015) and MPTP-induced Parkinson’s disease (PD; Chung et al., 2012) through anti-inflammatory and anti-immune processes.

Regulatory T cells (Tregs) play pivotal roles in suppressing systemic effector immune responses and maintaining immune tolerance (Baruch et al., 2015). The Tregs population is decreased in both the Aβ-induced (Zhang et al., 2015) and triple-transgenic AD mouse brains (Ye et al., 2016). The lowest percentages of all Tregs populations are also seen in patients with severe AD, and provide evidence of an inflammatory origin for AD (Saresella et al., 2010). In addition, the NF-κB complex, especially the REL and p65 (also known as RELA) subunits, are involved not only in promoting inflammation, but also in driving the Treg development by promoting the formation of forkhead box P3 (Foxp3)-specific enhanceosomes (Grinberg-Bleyer et al., 2018). Bee venom PLA2 (bv-sPLA2) has been reported to increase the Treg population *via* the CD206 receptor expressed in dendritic cell membranes (Kim et al., 2015), as well as suppress microglial activation *via* the modulation of Treg-mediated peripheral immune tolerance (Ye et al., 2016). In the present study, we investigated whether bv-sPLA2 alleviates LPS-induced inflammatory and immune responses and memory impairment, as well as the associated mechanisms, both *in vivo* and *in vitro*.

## Materials and Methods

### Materials

Bee venom sPLA2 was obtained from Inistst Company Limited (Gyeonggi-do, South Korea), dissolved in phosphate-buffered saline (PBS; final concentration: 1 mg/mL) and stored at −20°C until use. The LPS was purchased from Sigma-Aldrich (serotype 0111:B4; Sigma-Aldrich, St. Louis, MO, USA) and was prepared and stored as previously described (Han et al., 2019).

### Animals Experiments and Housing Conditions

**Figure 1:**
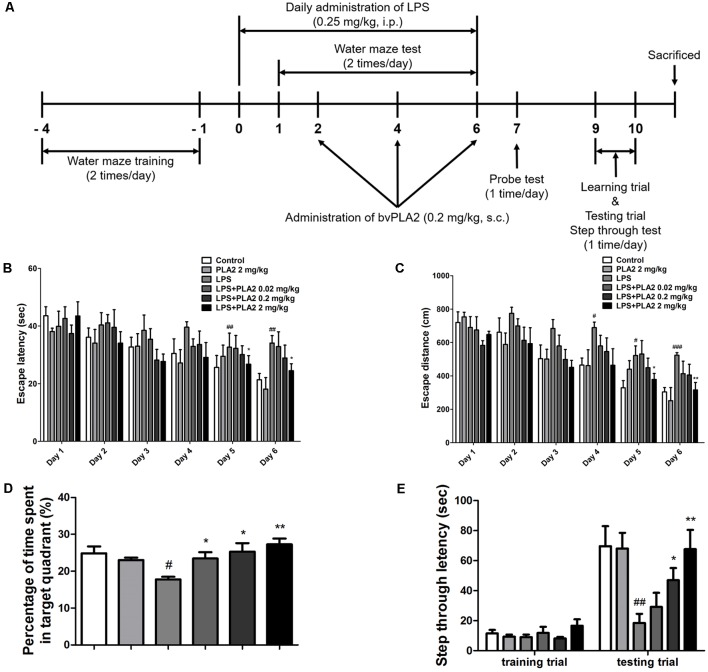
Effects of bv-sPLA2 on lipopolysaccharide (LPS)-induced improvement of memory impairment in the mice. **(A)** The mice (*n* = 8) were daily treated bv-sPLA2 by i.p. injection at dose of 0.02, 0.2 and 2 mg/kg for three times. Intraperitoneal injection of LPS (250 μg/kg) was treated except for control group for 7 days, and they were evaluated for learning and memory of spatial information using the water maze. **(B)** Escape latency, the time required to find the platform and **(C)** escape distance, the distance swam to find the platform was measured. After the water maze test, **(D)** probe test to measure maintenance of memory was performed. The time spent in the target quadrant and target site crossing within 60 s was represented. **(E)** A passive avoidance test was performed by step-through method. *n* = 8 per group. The data are shown as the means ± standard deviation (SD) of the mean. ^#^*p* < 0.05, ^##^*p* < 0.005, ^###^*p* < 0.001 control group vs. LPS group, *p < 0.05, **p < 0.005 LPS-group vs. LPS with bv-sPLA2 group.

Eight-week-old male imprinting control region (ICR) mice weighing 25 ± 2 g were purchased from Daehan Bio Link (Eumsung, South Korea). The experiment was carried out in accordance with the guidelines set out by the Chungbuk National University Animal Care Committee (CBNUA-1126-18-01). Mice were maintained and managed as previously described (Han et al., 2019). All mice had 7 days of adaptation before receiving 4 days of water maze training (Figure 1A). After the training, all the mice were randomly divided into six groups, as follows (*n =* 8/group): control group, 2 mg/kg bv-sPLA2 group, LPS group, LPS + 0.02 mg/kg bv-sPLA2 group, LPS + 0.2 mg/kg bv-sPLA2 group, and LPS + 2 mg/kg bv-sPLA2 group. The bv-sPLA2, dissolved in saline, was administered three times by intraperitoneal (i.p.) injection. Except for the control group, LPS (250 μg/kg) was administered daily to all groups for 7 days. Control mice were administered an equal volume of vehicle. Concurrent with bv-sPLA2/LPS treatment, behavioral tests for the evaluation of learning and memory capacity were performed using water maze, probe, and passive avoidance tests. Mice were euthanized after the behavioral tests by CO_2_ asphyxiation.

### Morris Water Maze

The water maze test is a widely accepted method for examining cognitive function and was performed according to Morris (1984). The maze test was performed using the SMART-CS (Panlab, Barcelona, Spain) program and equipment. A circular plastic pool (height: 35 cm, diameter: 100 cm) was filled with squid ink water kept at 22–25°C. An escape platform (height: 14.5 cm, diameter: 4.5 cm) was submerged 1–1.5 cm below the surface of the water in position. The test was performed two times a day for 6 days during the acquisition phase, with two starting points of rotational starts. The position of the escape platform was kept constant. Each trial lasted for 60 s or ended as soon as the mouse reached the submerged platform. Escape latency and escape distance of each mouse were monitored by a camera above the center of the pool connected to a SMART-LD program (Panlab, Barcelona, Spain). A quiet environment, consistent lighting, constant water temperature and a fixed spatial frame were maintained throughout the experimental period.

### Probe Test

The probe test was performed 1 day after the completion of the water maze test to assess memory consolidation. After removing the platform from the pool used in the water maze test, the mice were allowed to move freely and the probe test lasted for 60 s, as previously described (Han et al., 2019).

### Passive Avoidance Test

The passive avoidance response was determined using a “step-through” apparatus (Med Associates Inc., Vermont, USA); the apparatus is divided into an illuminated and a dark compartment (each 20.3 × 15.9 × 21.3 cm) joined through a small gate with a grid floor consisting of 3.175 mm stainless steel rods set 8 mm apart. A training trial was performed 48 h after the probe test. Mice were placed in the illuminated compartment facing away from the dark compartment for the training trial. When the mice had completely moved into the dark compartment, they received an electric shock (0.45 mA, 3-s duration) and were subsequently returned to their cage. After 24 h, each mouse was placed in the illuminated compartment and the latency to enter the dark compartment, defined as “retention,” was measured. The time taken for the mice to enter the dark compartment was recorded and described as step-through latency. The cut-off time limit of the retention trials was set at 180 s.

### Collection and Preservation of Brain Tissue

Following the completion of all the behavioral tests, the mice were euthanized with CO_2_ gas and then perfused with PBS. The brain was immediately removed from the skull of each mouse, separated into left and right brain, and randomly allocated either for storage at −80°C or fixation in a 4% formalin solution for 3 days at room temperature. Formalin-fixed brain tissues were embedded in paraffin and sectioned (8 μm).

### Microglial BV-2 Cell Culture

Microglial BV-2 cells were obtained from the American Type Culture Collection (Rockville, MD, USA) and maintained as previously described (Han et al., 2019). The cells were treated with several concentrations of bv-PLA2 (0.01, 0.1, or 1 μg/ml) for 3 h before LPS (1 μg/ml) treatment and harvested after 24 h.

### Western Blot Analysis

The hippocampus was separated from the brain tissue and homogenized and lysed by adding a protein extraction solution (PRO-PREP, iNtRON, Sungnam, South Korea). After measuring the total protein concentration using Bradford reagent (Bio-Rad, Hercules, CA, USA), western blotting was performed as previously described (Han et al., 2019). Protein-specific primary antibodies were purchased from Novus Biologicals (anti-APP; Littleton, CO, USA), Santa Cruz Biotechnology (anti-GFAP and anti-β-actin; Dallas, TX, USA), Cell Signaling Technology (anti-iNOS, anti-COX-2, anti-p65, and anti-p50; Danvers, MA, USA), and Abcam [anti-BACE1, anti-ionized calcium-binding adapter molecule 1 (IBA-1), and anti-Foxp3; Cambridge, MA, USA]. Secondary antibodies were purchased from Santa Cruz Biotechnology (anti-mouse, anti-rabbit, and anti-goat).

### Immunohistochemistry

Immunohistochemistry was performed as previously described (Lee et al., 2019). To detect target proteins, protein-specific primary antibodies were purchased from Novus Biologicals (anti-APP), Santa Cruz Biotechnology (anti-GFAP and anti-β-actin), Cell Signaling Technology (anti-iNOS and anti-COX-2), and Abcam (anti-BACE1, anti-IBA-1, and anti-Foxp3). Secondary antibodies were purchased from Vector Laboratories (anti-rabbit; Burlingame, CA, USA) and Santa Cruz Biotechnology (anti-goat).

### Measurement of Aβ_1–42_

Brain tissue lysates were obtained by adding protein extraction buffer containing protease inhibitor. The levels of Aβ_1–42_ in the tissue lysates containing 100 μg of protein were determined using a mouse amyloid-beta peptide-specific 1–42 ELISA kit (CUSABIO, Carlsbad, CA, USA), according to the manufacturer’s instructions.

### Assay of Beta-Secretase Activities

Beta-secretase activity in mouse brains and cultured BV-2 cells was determined using a beta-secretase activity assay kit (Abcam). Solubilized membranes were extracted from brain tissues or BV-2 cells with extraction buffer, incubated on ice for 1 h, and centrifuged at 5,000× *g* for 10 min at 4°C. The total protein concentration of the supernatant was measured using Bradford reagent (Bio-Rad), while beta-secretase activity in tissues or cell lysates containing 100 μg of protein were determined according to the manufacturer’s instruction. Fluorescence was read at excitation and emission wavelengths of 335 and 495 nm, respectively, using a fluorescence spectrometer (Gemini EM, Molecular Devices, San Jose, CA, USA).

### Statistical Analysis

Data were analyzed using GraphPad Prism version 4.03 software (GraphPad Software, La Jolla, CA, USA). Data are presented as means ± standard deviation (SD). For all data, differences were evaluated by one-way analysis of variance. When significant differences were found, these were compared using Tukey’s test. A value of *p* ≤ 0.05 was considered significant.

## Results

### bv-sPLA2 Treatment Improves Memory Function in LPS-Injected Mice

We investigated whether bv-sPLA2 treatment could ameliorate memory deficits in an LPS-induced mouse model of memory impairment. Mice received daily LPS injections [i.p.; 250 μg/kg for 7 days (day 0–6)] and three bv-sPLA2 injections [i.p.; 0.02, 0.2, and 2 mg/kg (days 2, 4, and 6); Figure 1A]. The water maze test is a widely used method for assessing memory and can be used to evaluate spatial learning and memory retention. To investigate the memory-ameliorating effects of bv-sPLA2, we carried out water maze and passive avoidance tests. The ability to learn and recall spatial memory was assessed through escape latency and distance using a water maze test lasting for 6 days (day 1–6). The mean escape latency and escape distance on the final day of the test were approximately 24.64 s and 352.61 cm, respectively, in the control group. Mice treated with LPS exhibited a mean escape latency and distance to the hidden platform of approximately 37.00 s and 502.79 cm, respectively. The mean escape latency and distance decreased to 35.39, 31.72, and 27.41 s; and 438.29, 415.87, and 387.50 cm with bv-sPLA2 treatment at the concentrations of 0.02, 0.2, and 2 mg/kg, respectively (Figures 1B,C). The day after the completion of the water maze test (day 7), a probe test was performed to calculate the time spent in the target quadrant zone as a test for memory retention. The mean time spent in the target quadrant was decreased in the LPS-treated mice (17.79%) compared to that in the control group (24.83%); however, administration of bv-sPLA2 to the memory-impaired mice increased the average time spent in the target quadrant to 23.44%, 25.33%, and 27.29% in the 0.02, 0.2, and 2 mg/kg treatment groups, respectively (Figure 1D). Our results suggest that LPS-treated mice spent more time looking for the hidden platform and exhibited fewer platform crossings than the control group, whereas the bv-sPLA2-treated mice spent less time looking for the platform and presented increased platform crossings compared to the group treated only with LPS. To evaluate memory-retention capacity, we performed a passive avoidance test (days 9 and 10). Although no significant differences were observed among the groups in the training trial, step-through latency was decreased to 18.45 s in the LPS-treated mice compared to that in the control group (69.63 s). The step-through latency for the LPS-treated mice recovered to 38.29 s, 46.92 s, and 67.59 s with bv-sPLA2 treatment at 0.02, 0.2, and 2 mg/kg, respectively (Figure 1E). Thus, our results indicated that bv-sPLA2 administration ameliorates LPS-induced memory impairment.

### bv-sPLA2 Inhibits Aβ Deposition in the LPS-Injected Mouse Brain

**Figure 2:**
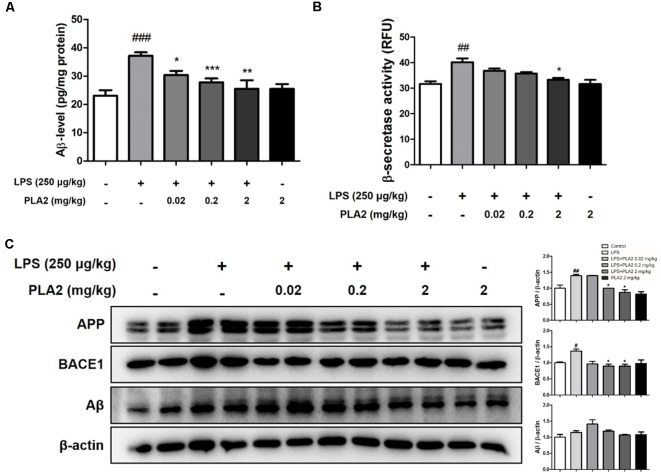
Effects of bv-sPLA2 on LPS-induced amyloid β (Aβ) deposition and expression of amyloidogenic protein in the mice brain. **(A)** The levels of Aβ_1–42_ in the brain of mice were assessed using a specific Aβ ELISA, *n* = 4 per group. **(B)** The β-secretase activity in the brain of mice was measured using assay kit, *n* = 4 per group. **(C)** The expression of amyloid precursor protein (APP) and BACE1 were detected by western blot using specific antibodies in the brain of mice. β-actin protein was used as an internal control. The data are shown as the means ± SD of the mean. ^#^*p* < 0.05, ^##^*p* < 0.005, ^###^*p* < 0.001 control group vs. LPS group, *p < 0.05, **p < 0.005, ***p < 0.001 LPS-group vs. LPS with bv-sPLA2 group.

Accumulation of Aβ may be related to memory dysfunction. Consequently, we measured Aβ deposition and beta-secretase activity in the mouse brain to determine whether there was an association between bv-sPLA2-mediated memory improvement and Aβ production. The Aβ levels in the brains of LPS-injected mice were higher than those in the control group, whereas, they were decreased in the brains of bv-sPLA2-treated mice (Figure 2A). The activity of beta-secretase was also higher in the brains of LPS-injected mice than in the control group but was decreased in the brains of bv-sPLA2-treated mice (Figure 2B). To confirm whether bv-sPLA2 can inhibit amyloidogenesis in the brain, APP and BACE1 protein expression were examined by western blot. The expression levels of APP, BACE1, and Aβ were increased in the brains of LPS-injected mice, but this was reversed by bv-sPLA2 administration (Figure 2C, Supplementary Figure S1).

### bv-sPLA2 Inhibits Neuroinflammation and NF-κB Activation in the LPS-Injected Mouse Brain

**Figure 3:**
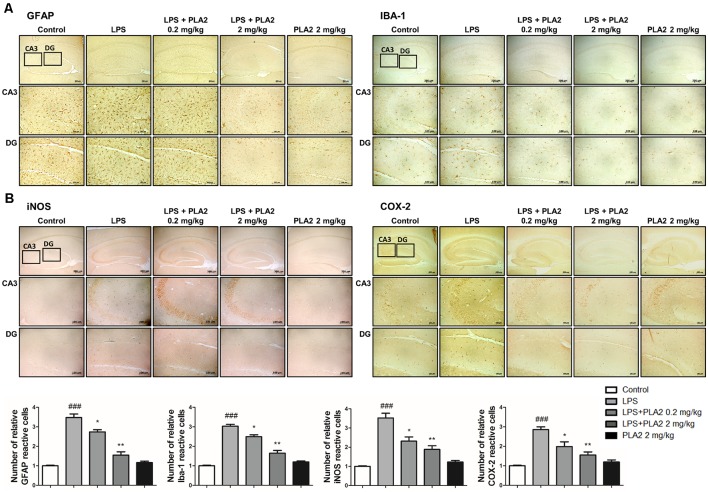
Effects of bv-sPLA2 on LPS-induced neuroinflammation in the mice brain. Immunohistochemical analysis of **(A)** GFAP, IBA-1 **(B)** iNOS and COX-2 antibodies were investigated two different regions (CA3; cornu ammonis 3 and DG; dentate gyrus) in 10-μm-thick sections of the brain hippocampus of mice with specific primary antibodies and the biotinylated secondary antibodies (scale bars, 100 μm). ^###^*p* < 0.001 control group vs. LPS group, *p < 0.05, **p < 0.005 LPS-group vs. LPS with bv-sPLA2 group.

**Figure 4:**
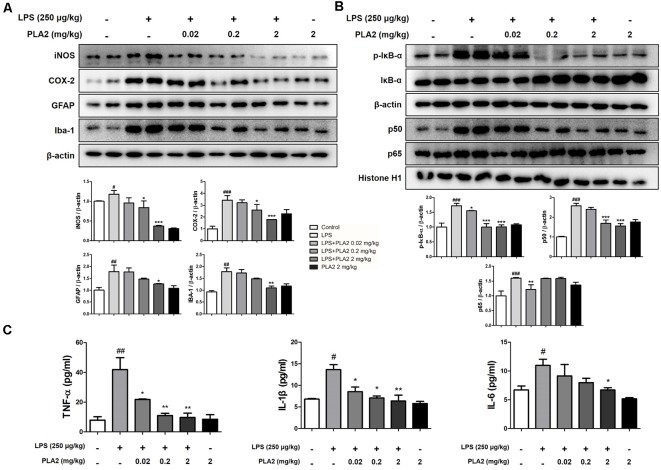
Effects of bv-sPLA2 on LPS-induced inflammatory responses and pro-inflammatory cytokines in the mice brain. **(A)** The expression of GFAP, IBA-1, iNOS and COX-2 were detected by western blot using specific antibodies in the brain of mice. **(B)** The expression of p-IκB-α, IκB-α, p50 and p65 were detected by western blot using specific antibodies in the brain of mice. β-actin protein was used as an internal control. **(C)** mRNA expression levels of pro-inflammatory cytokines such as tumor necrosis factor (TNF)-α, IL-1β and IL-6 in the brain of mice were measured using quantitative real-time RT-PCR. The data are shown as the means ± SD of the mean. ^#^*p* < 0.05, ^##^*p* < 0.005, ^###^*p* < 0.001 control group vs. LPS group, *p < 0.05, **p < 0.005, ***p < 0.001 LPS-group vs. LPS with bv-sPLA2 group.

Neuroinflammation is critical for Aβ generation, while astrocytes and microglial cells are important contributing factors for neuroinflammation and amyloidogenesis. To determine the effects of bv-sPLA2 on astrocyte and microglial cell activation, as well as on neuroinflammation, immunohistochemistry and western blot analysis were performed on the mouse brain to detect the expression of GFAP (an astrocyte marker protein), IBA-1 (a microglial marker protein), and inflammation-related proteins (iNOS and COX-2). In mice from the LPS-injected group, the number of GFAP- and IBA-1-reactive cells was higher than that in the control mice. However, the level of GFAP-reactive cells was lower in the bv-sPLA2-treated mouse brain compared to LPS treatment (Figure 3A). The number of iNOS and COX-2-reactive cells was also lower in the bv-sPLA2-treated mouse brain compared to that with LPS treatment (Figure 3B). In agreement with the immunohistochemistry results, the expression levels of GFAP, IBA-1, iNOS, and COX-2 were increased in the LPS-injected mice; however, these levels were dose-dependently decreased with bv-sPLA2 administration (Figure 4A, Supplementary Figure S2). The activity of the NF-κB complex has been implicated in amyloidogenesis and neuroinflammation since it regulates the expression of several genes involved in both these processes. Thus, we determined the activation state of NF-κB through the detection of IκBα phosphorylation and p50 (also known as NFκB1) and p65 nuclear translocation. Phosphorylation of IκBα and p50 and p65 nuclear translocation were both significantly decreased by bv-sPLA2 administration in a dose-dependent manner (Figure 4B, Supplementary Figure S3). The levels of the pro-inflammatory cytokines, TNF-α, IL-1β, and IL-6, were also elevated in the LPS-injected mouse brain. In contrast, these cytokine levels were dose-dependently reduced in the brains of bv-sPLA2-treated mice (Figure 4C).

### bv-sPLA2 Modulates Tregs Infiltration in the LPS-Injected Mouse Brain

**Figure 5:**
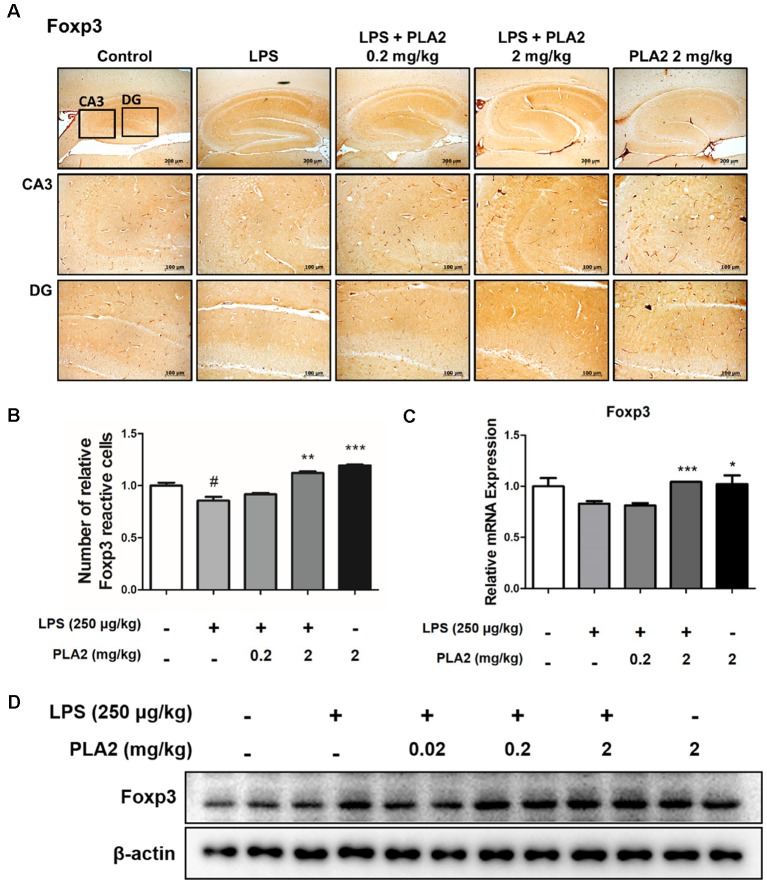
Effects of bv-sPLA2 on modulation of Tregs in the mice brain. **(A)** Immunohistochemical analysis of Foxp3 antibodies was investigated in two different regions (CA3; cornu ammonis 3 and DG; dentate gyrus) in 10-μm-thick sections of the brain hippocampus of mice with specific primary antibodies and the biotinylated secondary antibodies (scale bars, 100 μm). **(B)** Graph represented the relative intensity of Foxp3-reactive cells, *n* = 3 per group. **(C)** mRNA expression levels of Foxp3 in the brain of mice were measured using quantitative real-time RT-PCR. The data are shown as the means ± SD of the mean. ^#^*p* < 0.05 control group vs. LPS group, *p < 0.05, **p < 0.005, ***p < 0.001 LPS-group vs. LPS with bv-sPLA2 group. **(D)** The expression of Foxp3 was detected by western blot using specific antibody in the brain of mice. β-actin protein was used as an internal control.

To investigate whether bv-sPLA2 modulates Treg infiltration into the brains of LPS-injected mice, we performed immunohistochemistry to detect the expression of Foxp3 (a Treg marker protein) in the brains of LPS-treated mice. With LPS treatment, the number of Foxp3-reactive cells was lower than that in control mice. However, the number of Foxp3-reactive cells was increased in the bv-sPLA2-treated mouse brain compared to that in LPS-injected mice (Figures 5A,B). The mRNA level and protein level of Foxp3 were lower in the LPS-injected mouse brain compared to the control group. However, the Foxp3 mRNA and protein levels were higher in the bv-sPLA2-treated mouse brain compared to that in LPS-injected mice (Figures 5C,D, Supplementary Figure S4).

### bv-sPLA2 Reduces Amyloidogenesis and Neuroinflammation in Microglial BV-2 Cells

**Figure 6:**
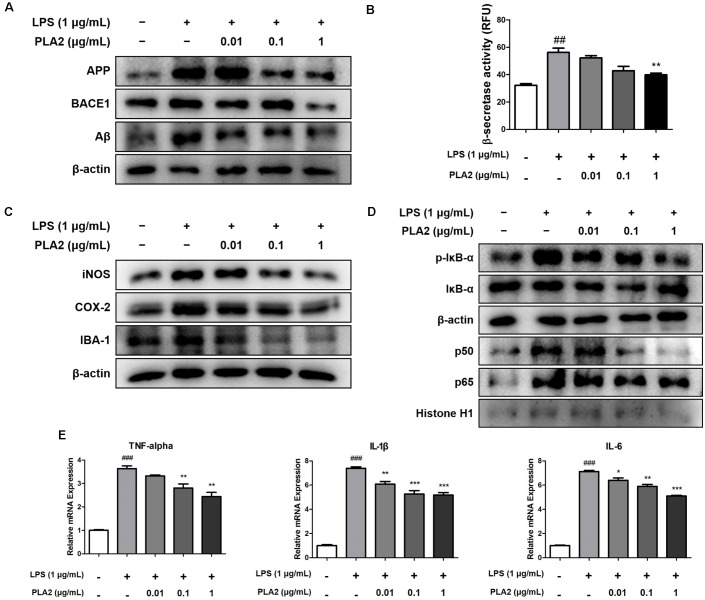
Effects of bv-sPLA2 on LPS-induced amyloidogenesis and neuroinflammation in the microglial cells. Microglia BV-2 cells were treated with LPS (1 μg/ml) and bv-sPLA2 (0.01, 0.1 and 1 μg/ml). **(A)** The expression of APP and BACE1 were detected by western blot using specific antibodies in the microglia BV-2 cells. **(B)** The levels of β-secretase activity in the microglia BV-2 cells were assessed using assay kit. **(C)** The expression of iNOS, COX-2 and IBA-1 were detected by western blot using specific antibodies in the microglia BV-2 cells. **(D)** The expression of p-IκB-α, IκB-α, p50 and p65 were detected by western blot using specific antibodies in the microglia BV-2 cells. β-actin protein was used as an internal control. **(E)** mRNA expression levels of pro-inflammatory cytokines such as TNF-α, IL-1β and IL-6 in the microglia BV-2 cells were measured using quantitative real-time RT-PCR. The data are shown as the means ± SD of the mean. ^##^*p* < 0.005, ^###^*p* < 0.001 control group vs. LPS group, *p < 0.05, **p < 0.005, ***p < 0.001 LPS-group vs. LPS with bv-sPLA2 group.

Microglia are the primary LPS-responsive cells in the CNS, and their activation is associated with neuroinflammation and amyloidogenesis during AD progression. To elucidate the anti-inflammatory roles of bv-sPLA2 in LPS-induced neuroinflammation, we performed western blot analysis, a beta-secretase activity assay, and real-time PCR on microglial BV-2 cells. The expression levels of APP and BACE1 were increased in the LPS-treated microglial BV-2 cells, but these levels were decreased with bv-sPLA2 treatment (Figure 6A, Supplementary Figure S5). The activity of beta-secretase was also increased in the LPS-induced microglial BV-2 cells but was dose-dependently inhibited with bv-sPLA2 treatment (Figure 6B). The expression levels of iNOS, COX-2, and IBA-1 were increased with LPS treatment, whereas they were reduced in BV-2 cells treated with bv-sPLA2 (Figure 6C, Supplementary Figure S6). Phosphorylation of IκBα and p50 and p65 nuclear translocation were both significantly decreased with bv-sPLA2 treatment in a concentration-dependent manner (Figure 6D, Supplementary Figure S7). The production of pro-inflammatory cytokines, such as TNF-α, IL-1β, and IL-6, was also increased in LPS-treated microglial BV-2 cells, but these levels were reduced with bv-sPLA2 administration (Figure 6E).

### bv-sPLA2 Has a Combinatorial Effect With a NF-κB Inhibitor

**Figure 7:**
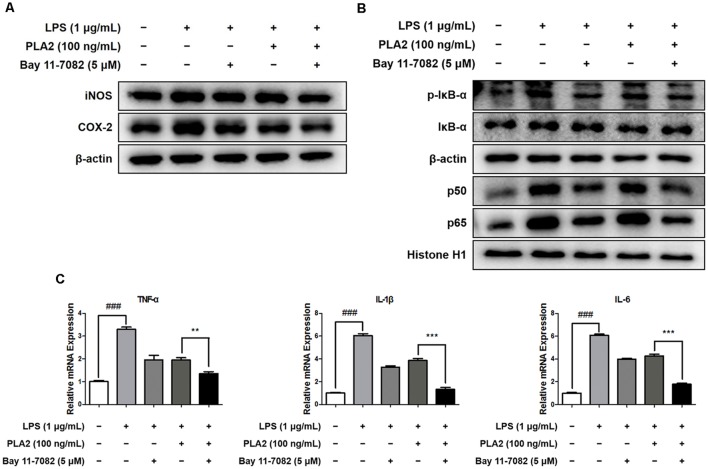
Combination effects of bv-sPLA2 and NF-κB inhibitor. Microglia BV-2 cells were treated with LPS (1 μg/ml), bv-sPLA2 (0.1 μg/ml) and Bay 11-7082 (5 μM). **(A)** The expression of iNOS and COX-2 was detected by western blot using specific antibodies in the microglia BV-2 cells. **(B)** The expression of p-IκB-α, IκB-α, p50 and p65 were detected by western blot using specific antibodies in the microglia BV-2 cells. β-actin protein was used as an internal control. **(C)** mRNA expression levels of pro-inflammatory cytokines such as TNF-α, IL-1β and IL-6 in the microglia BV-2 cells were measured using quantitative real-time RT-PCR. The data are shown as the means ± SD of the mean. ^###^*p* < 0.001 control group vs. LPS group, **p < 0.005, ***p < 0.001 LPS with bv-sPLA2 vs. LPS with bv-sPLA2 and Bay 11-7082 group.

To investigate the combinatorial effects of bv-sPLA2 and NF-κB inhibitor (Bay 11-7082, Sigma-Aldrich) treatment, we performed western blot and real-time PCR analyses on microglial BV-2 cells. Cells were treated with a combination of bv-sPLA2 (0.1 μg/ml) and the NF-κB inhibitor (5 μM), and whole-cell and nuclear lysates were extracted. The LPS-mediated increase in iNOS and COX-2 expression were significantly decreased with the combined treatment, compared to either treatment alone (Figure 7A, Supplementary Figure S8). In addition, IκBα phosphorylation, and p50 and p65 nuclear translocation, were inhibited significantly more by the combined treatment than either treatment alone (Figure 7B, Supplementary Figure S9). Co-treatment with bv-sPLA2 and the NF-κB inhibitor also attenuated the production of pro-inflammatory cytokines, including TNF-α, IL-1β, and IL-6 (Figure 7C). These data demonstrate that NF-κB inhibition exhibits greater anti-inflammatory activity than bv-sPLA2 alone.

## Discussion

Accumulating evidence has suggested that neuroinflammation is an important contributor to the pathophysiology of AD (Rubio-Perez and Morillas-Ruiz, 2012; Heneka et al., 2015a). Epidemiological studies have also revealed that anti-inflammatory drugs reduce the risk of developing AD (Szekely et al., 2004; Patel et al., 2005). Here, we demonstrated that bv-sPLA2 treatment alleviates LPS-induced amyloidogenesis and neuroinflammation, thereby suppressing memory impairment *via* inhibition of NF-κB, both *in vivo* and *in vitro*.

Our other studies have reported that systemic LPS treatment induces amyloidogenesis and neuroinflammation, which leads to impaired memory (Herber et al., 2004; Zarifkar et al., 2010; Choi et al., 2017a). Administration of LPS has been reported to induce Aβ generation, APP processing, and memory dysfunction (Lee et al., 2008). In our study, the expression of amyloidogenic and inflammatory proteins, like APP, BACE1, iNOS, and COX-2, increased with LPS treatment; however, administration of bv-sPLA2 decreased the expression of these proteins in both the LPS-treated brain and BV-2 cells. Activated astrocytes and microglial cells accelerate the progression of neuroinflammation, thus exacerbating neurodegenerative diseases, including AD (Cameron and Landreth, 2010). Activated astrocytes are known to upregulate BACE1 expression, thereby increasing Aβ generation (Wang et al., 2011). Activated astrocytes and microglial cells also release a series of cytokines, including TNF-α, IL-1β, and IL-6, that induce the production of Aβ (Quinn et al., 2003). Several studies have demonstrated that anti-neuroinflammatory compounds inhibit amyloidogenesis and ameliorate AD-related degeneration (Cameron and Landreth, 2010). We also previously found that treatment with the anti-neuroinflammatory agents, KRICT-9 (Lee et al., 2017), punicalagin (Kim et al., 2017), and matrine (Zhang et al., 2015), downregulated amyloidogenic proteins (APP, BACE1) and improved memory dysfunction. In the present study, we observed that bv-sPLA2 administration inhibited GFAP and IBA-1 expression, as well as LPS-induced cytokine release, in both the LPS-treated brain and BV-2 cells. These inhibitory effects on, both, neuroinflammation and activation of astrocytes and microglial cells, were associated with reduced amyloidogenesis and reduced LPS-induced memory impairment. Notably, Tregs play a significant protective role in AD. Depletion of Tregs aggravates cognitive decline in APP/PS1/tau triple transgenic (Baek et al., 2016) and APP/PS1 double transgenic mouse models of AD (Dansokho et al., 2013). Bee venom sPLA2 was reported to suppress microglial activation *via* the modulation of Treg-mediated peripheral immune tolerance (Ye et al., 2016). Adoptive transfer of Treg populations into APP/PS1/tau triple transgenic AD mice reduces the Aβ burden, microglial activation, and pro-inflammatory cytokine levels (Baek et al., 2016). In addition, Treg depletion in an APP/PS2 double-transgenic AD mouse model reduces the recruitment of activated microglia to Aβ deposits (Dansokho et al., 2013). In our model, bv-sPLA2 treatment increased the Treg population, an effect that was also associated with reduced microglial activation and Aβ accumulation. These data indicate that the anti-inflammatory and anti-immune responses, and inhibition of amyloidogenesis may be responsible for the memory-ameliorating effects of bv-sPLA2.

Neuroinflammation is one of the major pathological changes that occur in the AD brain and NF-κB signaling plays an important role in this condition (Chen C. H. et al., 2012; Badshah et al., 2016). Stimulation with LPS induces neuroinflammation through activation of NF-κB (Jung et al., 2007), and activation of NF-κB has been observed in neurons and astrocytes derived from AD patients (Kaltschmidt et al., 1997). The inhibitory effect of bv-sPLA2 on NF-κB may be associated with its anti-amyloidogenic effects (Bourne et al., 2007), and inhibition of NF-κB downregulates microglial responses to excitotoxic brain injury, resulting in a neuroprotective effect (Brambilla et al., 2005). Furthermore, the *APP* and *BACE1* promoters both contain functional NF-κB binding sites (Bourne et al., 2007; Chen C. H. et al., 2012). It was also reported that induction of NF-κB p65 expression increases BACE1 promoter activity and transcription, but that disruption of NF-κB p65 decreases BACE1 gene expression in p65 knockout cells (Chen C. H. et al., 2012). Additionally, several studies have reported that NF-κB inhibitors, like tetrandrine (He et al., 2011), salidroside (Gao et al., 2016), punicalagin (Kim et al., 2017), and an ethanol extract of *Nannochloropsis* *oceanica* (Choi et al., 2017b), can alleviate amyloidogenesis and neuroinflammation. Tregs directly inhibit NF-κB activation to repress pro-inflammatory cytokine gene expression (Bettelli et al., 2005). Tregs inhibit p65-mediated activation in HEK 293T cells overexpressing NF-κB p65 (Bettelli et al., 2005). In addition, the adoptive transfer of CD4^+^ CD25^+^ T cells into mice suppresses NF-κB activation in response to LPS treatment (O’Mahony et al., 2008). The transcriptional activity of NF-κB was also increased in Treg-deficient T cells (Bettelli et al., 2005). Infiltration of Tregs was shown to downregulate pro-inflammatory cytokines, such as TNF-α and IL-1β, but upregulate anti-inflammatory cytokines, such as IL-10, thereby ameliorating neuroinflammation after middle cerebral artery occlusion (Liesz et al., 2013). The major anti-inflammatory cytokine produced by Tregs, IL-10, inhibits the production of TNF-α, nitric oxide, and extracellular superoxide in microglial cells (Qian et al., 2010). In the present study, we demonstrated that bv-sPLA2 inhibits IκBα phosphorylation and translocation of p50 and p65 into the nucleus in LPS-injected mouse brains and LPS-induced microglial BV-2 cells. Furthermore, combined bv-sPLA2 and NF-κB inhibitor treatment inhibited iNOS and COX-2 expression, IκBα phosphorylation, and p50 and p65 nuclear translocation, compared to either treatment alone in the LPS-treated microglial BV-2 cells. Both iNOS and COX-2 are induced by inflammatory stimuli and play important roles in neuroinflammation (Chen et al., 2016). Recently, there have been reports that COX-1, an isoform of COX-2, plays a major role in neuroinflammation (Choi et al., 2009; Calvello et al., 2012, 2017). When combined, these data indicate that bv-sPLA2-mediated inhibition of NF-κB signaling may contribute to reducing amyloidogenesis and neuroinflammation, and improve memory function.

Bee venom is considered an effective therapy in several diseases, including rheumatoid arthritis and neurodegenerative and liver diseases (Zhang et al., 2018). Bee venom exerts an inhibitory effect in an LPS-induced mouse model of AD (Gu et al., 2015), rheumatoid arthritis (Darwish et al., 2013), PD (Kim et al., 2011), and growth of prostate cancer cells (Park et al., 2011) by regulating NF-κB activation. The main component in bee venom is melittin, followed by PLA2 (Billingham et al., 1973). Melittin binds directly to p50 and inhibits NF-κB activation in an LPS-induced mouse model of AD and microglial BV-2 cells (Moon et al., 2007; Gu et al., 2015). At a concentration of 0.2 mg/kg, bv-sPLA2 exerts greater neuroprotective effects than melittin at a concentration of 1.6 μg/kg for the same treatment period in an LPS-induced mouse model of AD; however, the Aβ level was more significantly inhibited in bv-sPLA2-treated mice (34.21%) than in those treated with melittin (16.67%; Gu et al., 2015). It was also reported that cisplatin-induced kidney injury in mice was alleviated by treatment for 5 days with bv-sPLA2 at a concentration of 0.2 mg/kg (Kim et al., 2015). In addition, 1-methyl-4-phenyl-1,2,3, 6-tetrahydropyridine-induced PD was alleviated by bv-sPLA2 treatment at a concentration of 0.2 mg/kg for 6 days (Chung et al., 2012). In the present study, bv-sPLA2 treatment given three times a week alleviated AD-like symptoms in a LPS-induced murine model of AD at the concentrations of 0.2 and 2 mg/kg. These results indicate that a low dose of bv-sPLA2 has potential for development as a drug candidate. LPS-induced models to investigate AD.

In conclusion, we demonstrated that bv-sPLA2 attenuates LPS-induced amyloidogenesis and neuroinflammation, thereby alleviating memory impairment *via* inhibition of NF-κB.

## Data Availability Statement

All datasets generated for this study are included in the article/Supplementary Material.

## Ethics Statement

The animal study was reviewed and approved by Chungbuk National University Animal Care Committee (CBNUA-1126-18-01).

## Author Contributions

HH, JHH and YL designed experiments, carried out most of the experiments, performed data analysis, and were the primary writers of the manuscript. KK, JY, SKK, YP and SHK contributed to preparation and performed the research. JTH supervised the entire project and had a major role in experimental design, data interpretation, and editing the manuscript before submission. All authors read and approved the final manuscript.

## Conflict of Interest

SHK was employed by the company INISTst Co. LTD.

The remaining authors declare that the research was conducted in the absence of any commercial or financial relationships that could be construed as a potential conflict of interest.
