# Supplementary material for: Bee Venom Soluble Phospholipase A2 Exerts Neuroprotective Effects in a Lipopolysaccharide-Induced Mouse Model of Alzheimer’s Disease via Inhibition of Nuclear Factor-Kappa B
Source: Front Aging Neurosci. 2019 Nov 1;11:287. doi: 10.3389/fnagi.2019.00287 (PMC6839038; doi:10.3389/fnagi.2019.00287)

Supplementary information for

Neuroprotective effects of bee venom soluble phospholipase A2 in the lipopolysaccharide-induced mouse model of Alzheimer's disease via inhibition of nuclear factor-kappaB

Hyeon Joo Ham[1#], Ji Hye Han[1#], Yong Sun Lee[1], Ki Cheon Kim[1], Jaesuk Yun[1], Se Hyun Kim[2], Jin Tae Hong[1*]

[1]College of Pharmacy and Medical Research Center, Chungbuk National University, Chungbuk, Republic of Korea

[2] INISTst Co., Gyeonggi, Republic of Korea

*Correspondence : Dr. Jin Tae Hong (jinthong@chungbuk.ac.kr), College of Pharmacy and Medical Research Center, Chungbuk National University, Chungbuk, Republic of Korea, Tel: +82-043-261-2813, Fax: +82-043-268-2732

[#] Hyeon Joo Ham and Ji Hye Han contributed equally to this work.

Hyeon Joo Ham: prodijoo0918@nate.com

Ji Hye Han: aff4434@naver.com

Yong Sun Lee: kallintz@gmail.com

Ki Cheon Kim: k.kicheon@gmail.com

Jaesuk Yun: jyun@chungbuk.ac.kr

Se Hyun Kim: sean0101@inistst.com

Supplementary Figure S1. Full images of electrophoretic blot in figure 2C

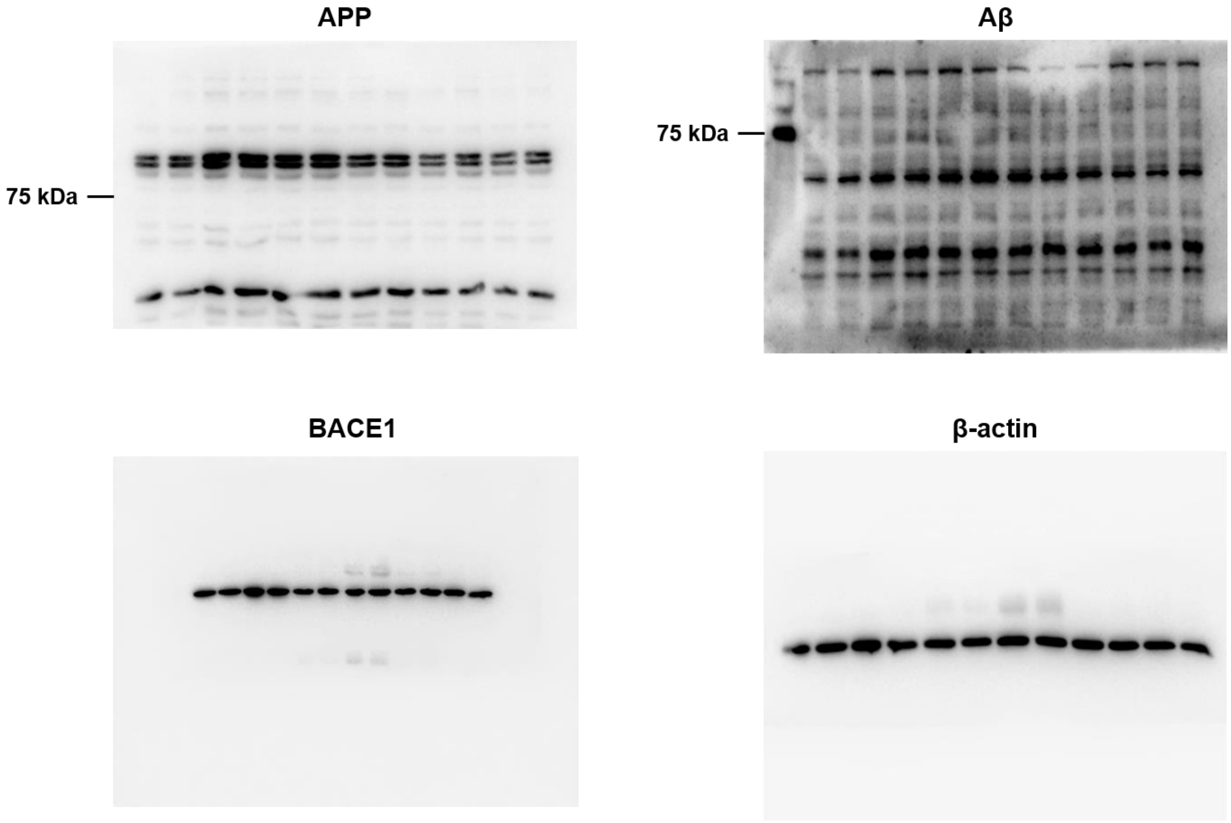

Supplementary Figure S2. Full images of electrophoretic blot in figure 4A

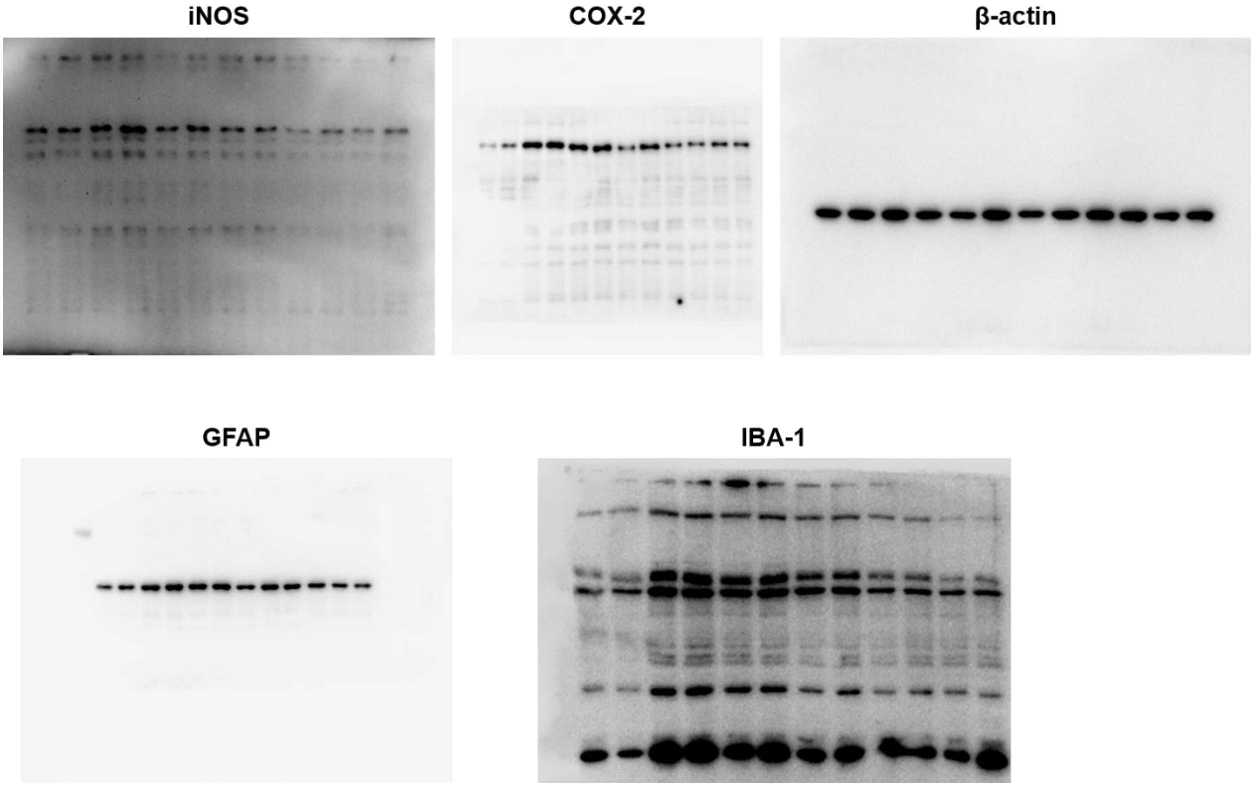

Supplementary Figure S3. Full images of electrophoretic blot in figure 4B

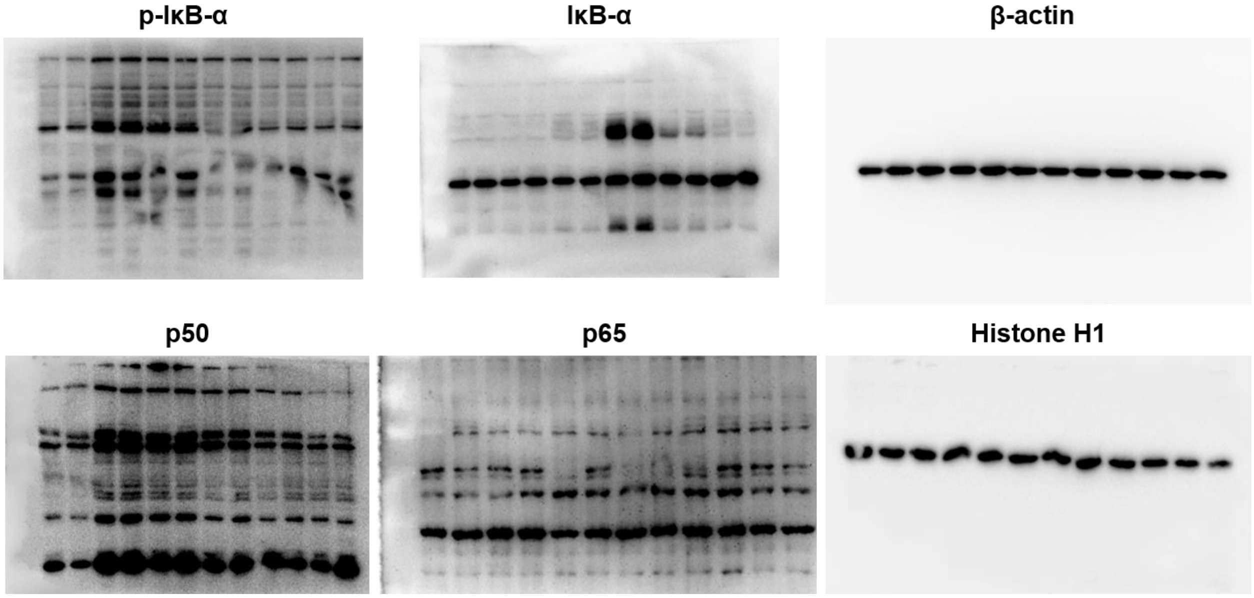

Supplementary Figure S4. Full images of electrophoretic blot in figure 5D

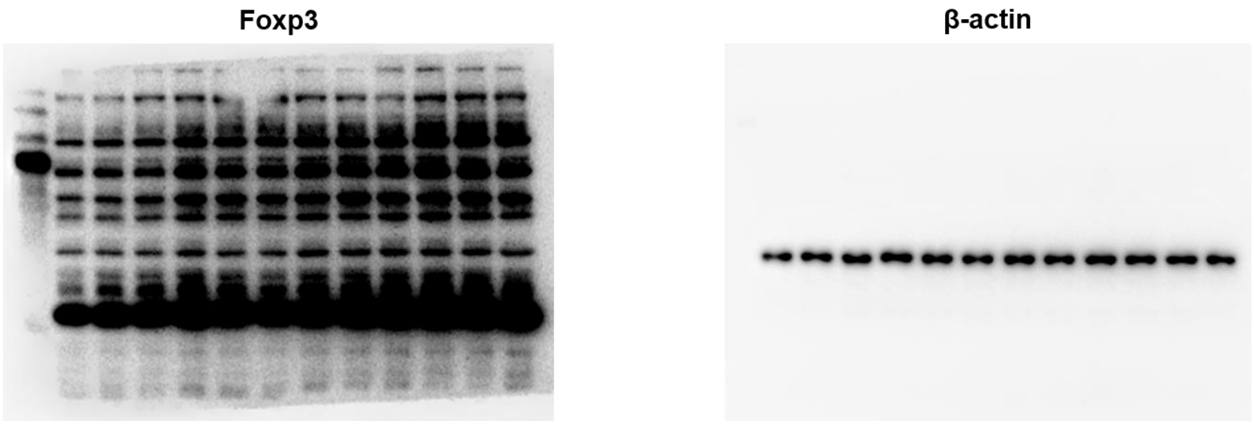

Supplementary Figure S5. Full images of electrophoretic blot in figure 6A

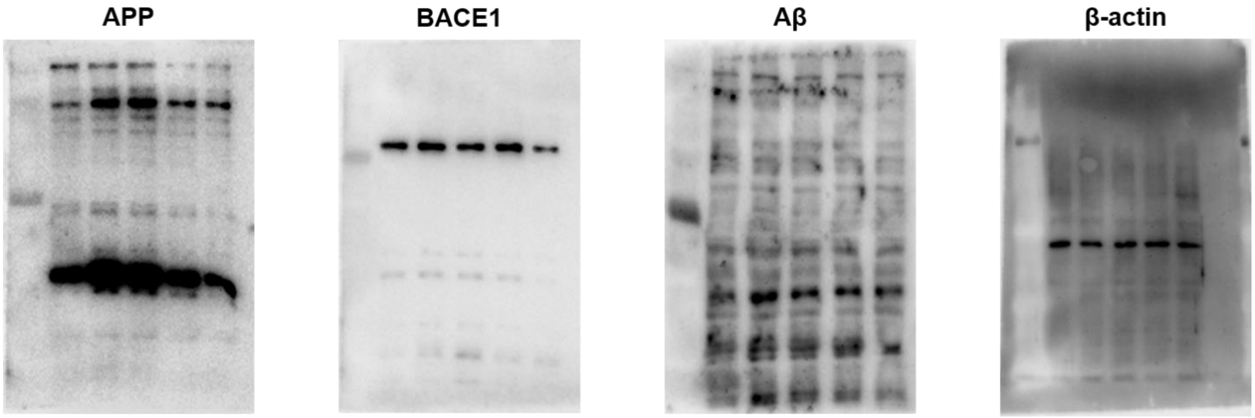

Supplementary Figure S6. Full images of electrophoretic blot in figure 6C

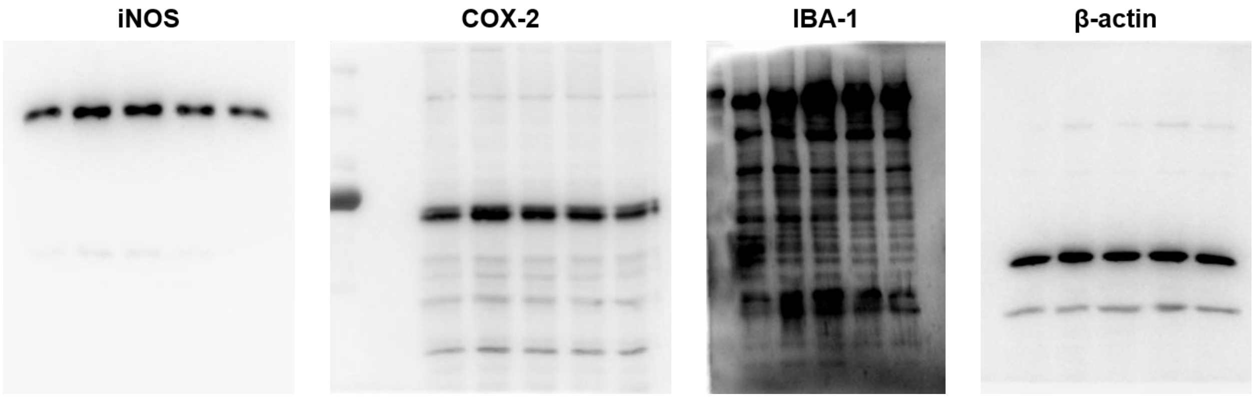

Supplementary Figure S7. Full images of electrophoretic blot in figure 6D

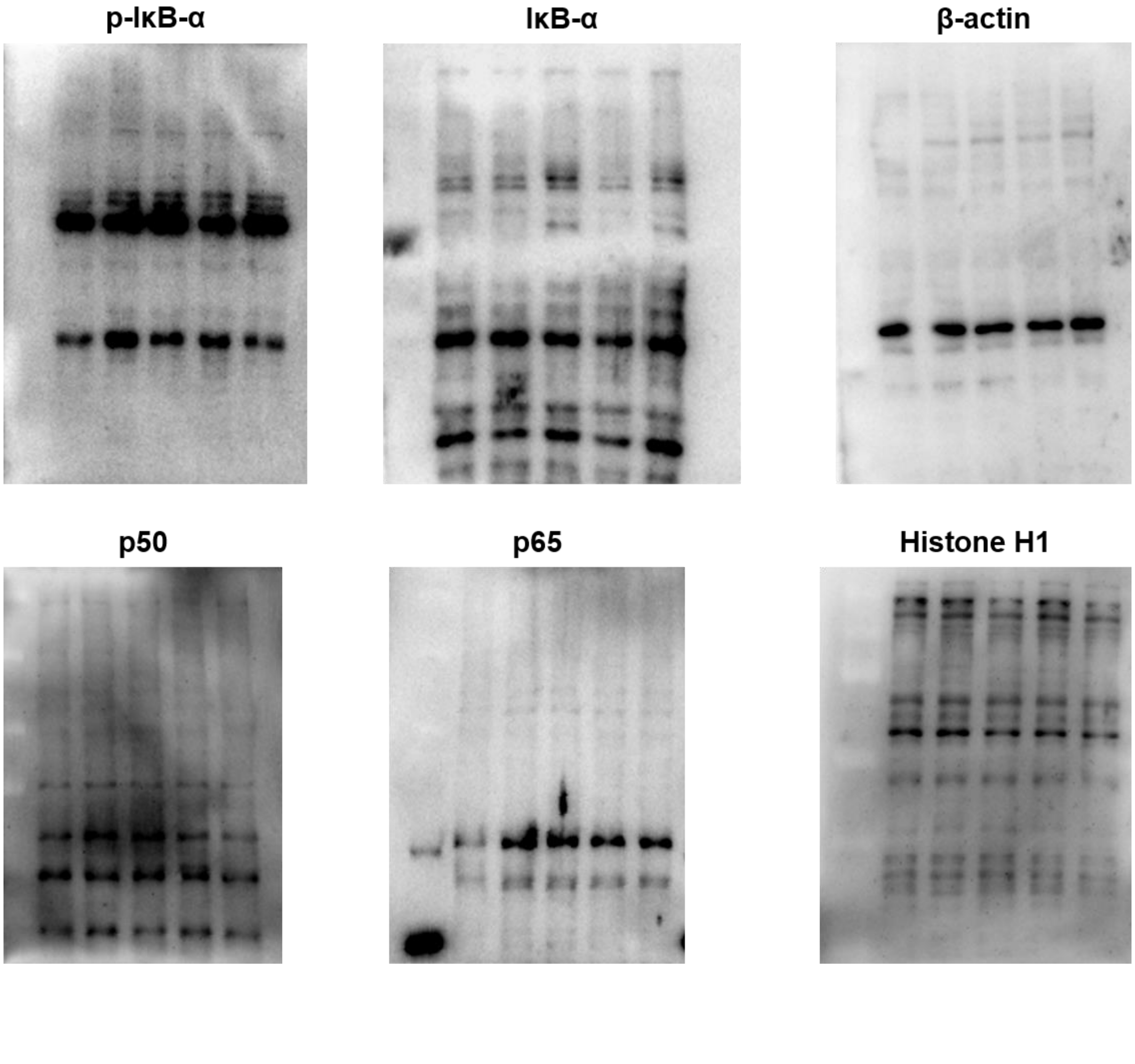

Supplementary Figure S8. Full images of electrophoretic blot in figure 7A

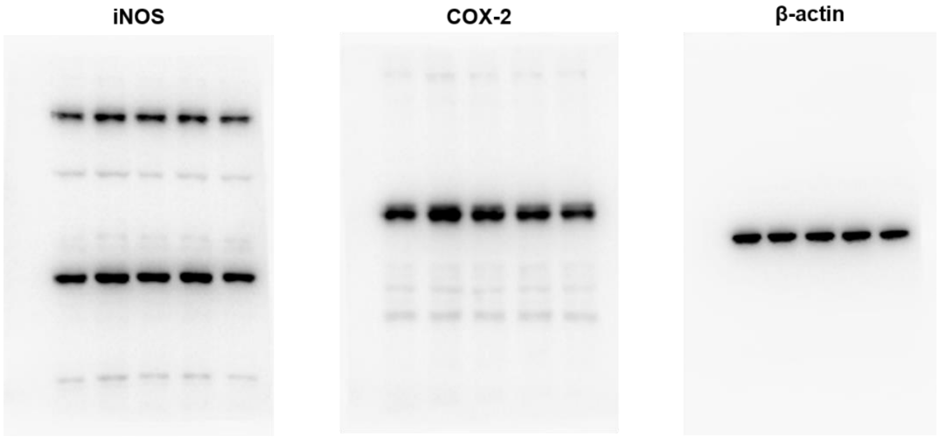

Supplementary Figure S9. Full images of electrophoretic blot in figure 7B

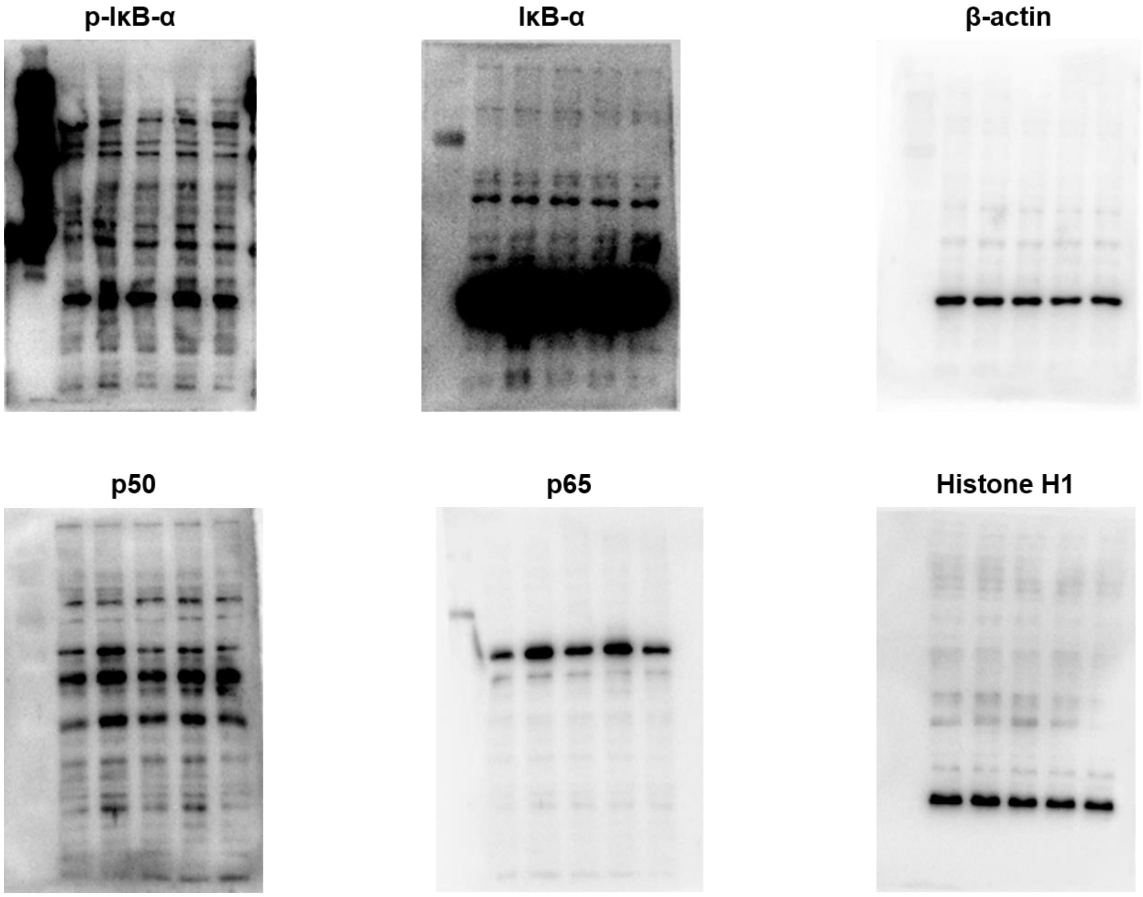

Supplement: Supplementary file 1 [file Data_Sheet_1.PDF]
